# Transarterial Chemoembolization of Hepatocellular Carcinoma with Oncozene Microspheres: An Initial, Short-Term Clinical Experience—A Retrospective, Matched, Comparison Study

**DOI:** 10.3390/life11070600

**Published:** 2021-06-23

**Authors:** Matthew L. Hung, Jerry Jiang, Harry Trieu, Frank Hao, Navid Eghbalieh, Peng-Xu Ding, Edward Wolfgang Lee

**Affiliations:** 1 Division of Interventional Radiology, Department of Radiology, UCLA Medical Center, David Geffen School of Medicine at UCLA, Los Angeles, CA 90095, USA; Mlhung17@gmail.com (M.L.H.); Jerrypjiang@gmail.com (J.J.); Harry.Trieu@gmail.com (H.T.); FrankHao@mednet.ucla.edu (F.H.); Navid.Eghbalieh@providence.org (N.E.); jieru375@sina.com (P.-X.D.); 2 Dumont-UCLA Transplant Center, Pfleger Liver Institute, Division of Liver and Pancreas Transplantation, Department of Surgery, David Geffen School of Medicine at University of California, Los Angeles, CA 90095, USA

**Keywords:** transarterial chemoembolization, HCC, Oncozene microspheres, LC beads

## Abstract

**Background:** The purpose of this study is to describe a single institution’s experience using Oncozene (OZ) microspheres for transarterial chemoembolization (OZ-TACE) of hepatocellular carcinoma (HCC), and to compare tolerability, safety, short-term radiographic tumor response, progression-free survival (PFS), and overall survival (OS) of these procedures to TACE (LC-TACE) performed with LC beads (LC). **Methods:** A retrospective, matched cohort study of patients undergoing DEB-TACE (drug-eluting bead transarterial chemoembolization) with OZ or LC was performed. The cohort comprised 23 patients undergoing 29 TACE with 75 or 100 μm OZ and 24 patients undergoing 29 TACE with 100–300 μm LC. Outcome measures were changes in liver function tests, complications, treatment tolerability, short-term radiographic tumor response according to modified RECIST criteria for HCC, PFS, and 1-year OS. The Mann–Whitney U test, Fisher exact test, and log rank test were used to compare the groups. **Results:** The BCLC or Child–Pugh scores were similar between the OZ and LC group. However, the two groups differed with respect to the etiology of background cirrhosis (*p* = 0.02). All other initial demographic and tumor characteristics were similar between the two groups. OZ-TACE used less doxorubicin per treatment compared to LC-TACE (median 50 vs. 75 mg; *p* = 0.0005). Rates of pain, nausea, and postembolization syndrome were similar, irrespective of the embolic agent used. OZ-TACE resulted in an overall complication rate comparable to LC-TACE (20.7% vs. 10.3%; *p* = 0.47). LC-TACE resulted in a higher percent increase in total bilirubin on post-procedure day 1 (median 18.8 vs. 0%; *p* = 0.05), but this difference resolved at 1 month. Both OZ-TACE and LC-TACE resulted in similar complete (31% vs. 24%) and objective (66% vs. 79%) target lesion response rates on 1-month post-TACE imaging. Both OZ-TACE and LC-TACE had similar median progression-free survival (283 vs. 209 days; *p* = 0.14) and 1-year overall survival rates (85% vs. 76%; *p* = 0.30). **Conclusion:** With a significantly reduced dose of doxorubicin, TACE performed with Oncozene microspheres in a heterogeneous patient population is well-tolerated, safe, and produces a similar radiological response and survival rate when compared to LC Bead TACE.

## 1. Introduction

Hepatocellular carcinoma (HCC) is the sixth most common cancer and the second most common cause of death from cancer worldwide [1]. HCC-related incidence and mortality in the United States are projected to increase in the next decade [2]. Transarterial chemoembolization (TACE) is the treatment of choice for HCC not amenable to surgical or ablative techniques in patients with well-compensated liver disease [3]. TACE can be performed using an ethiodized oil emulsion or drug-eluting beads (DEBs). The PRECISION V trial demonstrated that TACE performed with DEBs resulted in comparable response rates with reduced liver toxicity and drug-related adverse events when compared to TACE performed with ethiodized oil emulsion [4]. Since the publication of these results, the use of DEBs has become increasingly more common [5].

Oncozene microspheres (Varian, Palo Alto, CA, USA) are among the most recent commercially available DEBs. Theoretical advantages include the availability in small diameters (40, 75, and 100 μm), which could allow for more distal occlusion and denser packing in intratumoral vessels, maximizing local delivery of the chemotherapeutic while sparing normal liver parenchyma [6]. In vitro experiments and pharmacokinetics demonstrate sustained release of the chemotherapeutic compared to LC Bead (Boston Scientific, Marlborough, MA, USA) [7]. The size of the Oncozene microspheres is tightly calibrated; other embolic agents are constituted with a more heterogeneous distribution, which could be problematic as larger spheres could occlude vessels proximally and prevent the distal delivery of the smaller spheres. The smaller size of Oncozene may result in a larger quantity of embolics being delivered to the tumor.

Despite these theoretical benefits, the clinical use of Oncozene microspheres described in the literature is limited [8,9,10,11,12]. The purpose of this study is to assess the short-term radiographic tumor response, safety, and tolerability of DEB-TACE performed with Oncozene microspheres (OZ-TACE), and to compare these outcomes with those from DEB-TACE performed with LC beads (LC-TACE), a commonly used DEB agent. We hypothesized that OZ-TACE would result in higher rates of complete and partial response compared to LC-TACE, with less adverse effects.

## 2. Materials and Methods

This single-center study was approved by the Institutional Review Board at our hospital (IRB#21-000278) and is compliant with the Health Insurance Portability and Accountability Act, with waiver of informed consent. A retrospective, matched cohort analysis was conducted on 29 OZ-TACE for 23 patients and 29 LC-TACE for 24 patients. TACE procedures performed with LC beads were selected as a matched cohort with respect to patient age and tumor characteristics. All patients had biopsy-proven HCC or Liver Imaging Reporting and Data System (LI-RADS) 5 liver masses. Patients were considered to be eligible for TACE if they were not candidates for percutaneous or surgical ablative therapies, had well-compensated cirrhosis with a Child–Pugh score up to B, and had a patent main portal vein.

### 2.1. TACE Technique

Catheter access to the hepatic vasculature was obtained in a standard fashion. Superselective catheterization of feeding vessels was performed with a coaxial microcatheter system. After distal positioning of the microcatheter, the DEB agent was delivered. The delivered Oncozene microspheres were 75 μm in size (except in 5 procedures with 100 μm). All procedures with LC Bead used 100–300 μm beads. Doxorubicin was the chemotherapeutic agent in all procedures. Embolic preparations were administered until near-stasis and absence of tumoral enhancement on angiography. Real-time fluoroscopy was utilized to minimize the reflux into nontarget vessels. All procedures were performed by interventionalists with 10–25 years of experience. 

### 2.2. Post-Procedural Management and Surveillance

Following the procedure, patients were admitted for overnight observation. Patients were discharged the next day in the absence of serious adverse events and control of pain and nausea with oral medications. Data were collected regarding narcotic use during the postprocedural hospitalization, and a standardized opioid equivalency table was used to account for differences in potency and routes of administration [13]. Liver function tests were drawn on the morning of the procedure, post-procedure day 1, and at a scheduled clinic visit 1 month after TACE. 

Complications were noted in accordance with the quality improvement guidelines published by the Society of Interventional Radiology [14]. Major complications included those requiring therapy and minor hospitalization (48 h), those requiring major therapy with an unplanned increase in the level of care and prolonged hospitalization, permanent adverse sequelae, and death. Minor complications included those without clinical consequence and required nominal therapy at most. Treatment-related toxicity was also classified according to the National Cancer Institute Common Terminology Criteria for Adverse Events, version 4.03, where appropriate [15].

### 2.3. Radiographic Tumor Response

Imaging with either contrast-enhanced multiphasic computed tomography or magnetic resonance imaging with Eovist was obtained within 1 month prior and after TACE. Serial images assessing progression were subsequently obtained at regular intervals as determined by the managing interventional radiologist and/or oncologist. Objective tumor response was evaluated using the modified Response Evaluation Criteria in Solid Tumors (mRECIST) assessment for HCC [16,17,18]. Target lesions were at least 1 cm in diameter, suitable for repeat measurement, demonstrated intratumoral enhancement on imaging, and were treated with TACE. A maximum of two lesions satisfying these criteria with the greatest intratumoral enhancement were selected as the target lesions for each patient. All other arterially enhanced lesions were considered nontarget lesions. Blinded review of each patient’s imaging was conducted by two of the authors (M.L.H. and E.W.L.) in a consensus reading session.

### 2.4. Statistics

Significant differences between groups were analyzed with the Mann–Whitney U test and Fisher exact test, where appropriate. Kaplan–Meier analysis using the log-rank test was performed to assess survival differences. All statistical analyses were performed using SPSS Statistics 15 (IBM Corp., Armonk, NY, USA). A *p* < 0.05 was considered to be statistically significant.

## 3. Results

### 3.1. Baseline Clinical Characteristics

Our cohort consisted of 23 patients (15 males, 8 females, mean age 68 years) who underwent 29 OZ-TACE and 24 patients (15 males, 9 females, mean age 65 years) who underwent 29 LC-TACE. The mean age of the entire cohort was 67 years. The baseline clinical characteristics are summarized in Table 1. The two groups were balanced with respect to age, sex, history of previous treatment, Eastern Cooperative Oncology Group (ECOG) status, baseline alpha-fetoprotein (AFP), maximum tumor size and enhancement, tumor burden and distribution, Barcelona clinic liver cancer (BCLC) stage, and Child–Pugh score. 

The etiology of cirrhosis differed between the two groups, where patients who underwent OZ-TACE had a higher rate of nonalcoholic steatohepatitis and patients who had LC-TACE had a higher rate of hepatitis C and alcoholic cirrhosis (*p* = 0.02). Patients who had OZ-TACE had higher albumin levels (3.8 vs. 3.4, *p* = 0.008) and lower INR (1.1 vs. 1.2, *p* = 0.0001) at baseline compared to patients with LC-TACE. The range of doses prepared per treatment for both OZ-TACE and LC-TACE were similar at 75–100 mg. However, significantly less doxorubicin was delivered per treatment when Oncozene microspheres were used (an average dose of 50 in OZ-TACE vs. 75 mg in LC-TACE, *p* = 0.0005).

### 3.2. Clinical Toxicities and Adverse Events

During the postprocedural hospitalization, the observed rates of transient fever were not significantly different between the OZ-TACE and LC-TACE groups (17% vs. 3%, *p* = 0.09; Table 2). The proportion of patients who had pain (76% vs. 59%, *p* = 0.26), nausea (72% vs. 59%, *p* = 0.41), or postembolization syndrome (86% vs. 83%, *p* = 1) was also similar between the two groups (Table 2). Patients in the Oncozene group had a similar daily narcotic requirement compared to patients in the LC Bead group (15 vs. 7.5 mg of PO morphine, *p* = 0.10). 

Patients in the LC Bead group experienced a higher percent increase in total bilirubin on post-procedure day 1 compared to patients in the Oncozene group (18.8% vs. 0%, *p* = 0.05; Table 3), but this difference resolved in 1 month. There was a higher rise in INR 1 month after TACE in the OZ-TACE group compared to the LC Bead group, although the median percent change in both groups was 0 (*p* = 0.009). All other changes in laboratory values were mild, transient, and were similar between the two groups (Table 3). 

There were six complications (21%) in the OZ-TACE group and three complications (10%) in the LC-TACE group (*p* = 0.47), according to the Society of Interventional Radiology classification system. Complications in the Oncozene group were considered to be major: three patients required readmission or prolonged hospitalization for pain and nausea; two patients required readmission for fever and leukocytosis without an identified source of infection, which resolved with antibiotics; and one patient developed ischemic cholecystitis, requiring cholecystostomy tube placement. Of the three complications in the LC Bead group, two were major and one was minor: two patients required prolonged hospitalization for pain and nausea; and one patient was evaluated in the emergency department for pain and nausea, but did not require admission.

### 3.3. Radiographic Tumor Response

**Figure 1:**
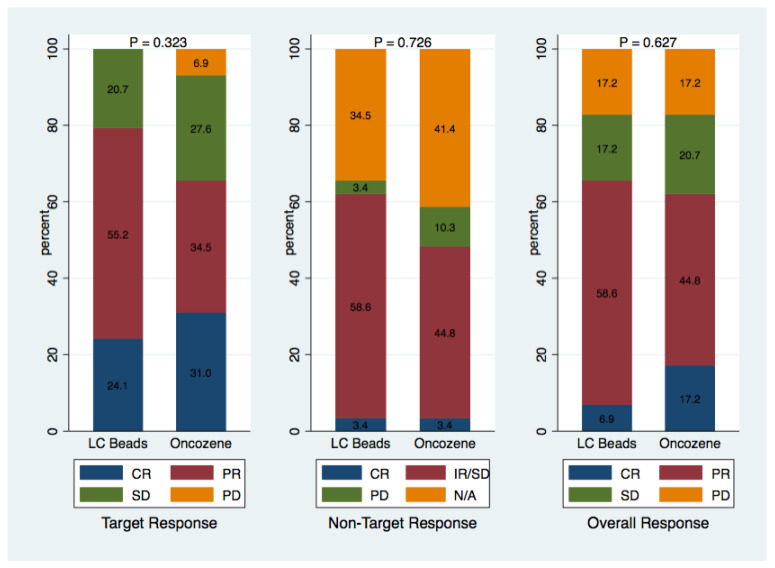
The graph shows the response to treatment 1 month following TACE, as measured by the modified Response Evaluation Criteria in Solid Tumors assessment for HCC. No significant differences were found between the Oncozene and LC Bead groups. CR = complete response; PR = partial response; SD = stable disease; IR = incomplete response; PD = progressive disease.

**Figure 2:**
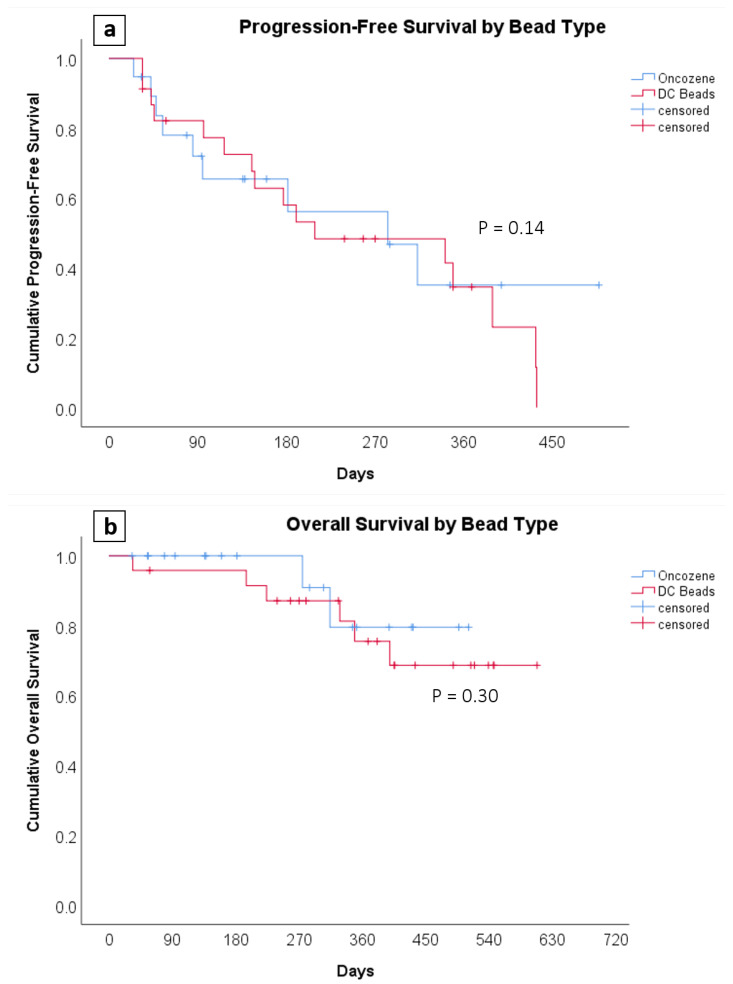
The graph shows the Kaplan–Meier survival curves of (**a**) progression-free and (**b**) overall survival between the Oncozene (blue) and LC Bead treatment groups (red). The median length of follow-up was 381 days. Both progression-free and overall survival were not significantly different between the two groups.

The use of Oncozene microspheres as compared to LC Bead resulted in similar complete (31% vs. 24%) and objective (66% vs. 79%) target lesion response rates on imaging 1 month after TACE (*p* = 0.32; Figure 1). Nontarget lesion response rates were also similar between the two groups. The choice of embolic agent did not impact the overall complete (17% vs. 7%) and objective (62% vs. 66%) response rates (*p* = 0.63; Figure 1). On long-term follow-up, the median follow-up time was 381 days (Interquartile Range: 239, 497). Median progression-free survival (283 vs. 209 days) did not differ significantly between the OZ-TACE and LC-TACE groups (*p* = 0.14). The 1-year overall survival rates were also similar (85% vs. 76%) (*p* = 0.30; Figure 2).

## 4. Discussion

The present study demonstrates that the choice between Oncozene microspheres and LC Bead during TACE did not significantly impact the complication rates or treatment tolerability. When the short-term radiographic response was assessed, the two groups demonstrated comparable rates of complete and objective target lesion response.

Although the distribution of cirrhosis etiology differed between the two groups, we would not expect this to affect our outcomes of safety, tolerability, and short-term radiographic response. Furthermore, Trevisani et al. demonstrated that the etiology of cirrhosis did not impact the survival of patients with HCC, with factors such as oncologic features and treatment found to be more important [19]. The difference in baseline serum albumin level and INR between the two groups was statistically significant. However, the differences in median albumin (3.8 vs. 3.4 g/dL) and INR (1.1 vs. 1.2) are unlikely to be clinically significant, and this is reflected in the similar distribution of Child–Pugh A/B patients between the two groups. These considerations, in conjunction with similar age, sex, and oncologic features, demonstrate that the groups are balanced for the purposes of our primary outcomes.

In our series, the choice of DEB did not impact the short-term radiographic response, progression-free survival, or overall survival. We hypothesized that Oncozene microspheres would perform better than 100–300 μm LC Bead on the basis of a few studies. First, Lee et al. demonstrated that upon histopathologic analysis of a rabbit tumor model, 100–300 μm particles were distributed intratumorally and at the periphery, while 300–500 μm particles were mainly distributed at the tumor periphery and within the hepatic artery at a distance from the tumor [6]. The clinical benefit of smaller particles was demonstrated in a study by Padia et al., where an EASL complete response rate of 59% was achieved with 100–300 μm particles as compared to 36% with 300–500 μm particles [20]. Interestingly, the same study showed that patients who were treated with 100–300 μm particles had a lower incidence of postembolization syndrome (8% vs. 36%) and fatigue (40% vs. 70%) compared to patients treated with 300–500 μm particles. There may be a critical particle size beyond which smaller diameters do not significantly prevent further intraparenchymal collateral flow or enhance local effects of the chemotherapeutic. The same argument could explain why patients in each group experienced similar amounts of pain and nausea; the smaller Oncozene microspheres may only marginally reduce global ischemia. Although the survival outcomes were similar between the two groups, many patients underwent additional local or systemic therapies following TACE, which could obscure any potentially isolatable survival differences between the two approaches.

Although Oncozene microspheres and LC Bead appeared to impact tumor radiographic response similarly in our study, it is worth noting that less doxorubicin was used per treatment when Oncozene microspheres were used. Of note, the average amount of doxorubicin prepared for each treatment was similar in both groups. This is likely due to in vivo pharmacokinetic data demonstrating a longer release time of chemotherapeutic and greater area under the concentration–time curve with Oncozene microspheres as compared to LC Bead [7]. Future investigation with dose titration studies could be valuable in identifying chemotherapy doses that maintain efficacy while minimizing biliary injury and anthracycline-related toxicity, with important implications for patients with cardiovascular comorbidities. Rates of cardiomyopathy range from 3–25% for cumulative doxorubicin dosages greater than 300 mg/m^2^, which is a significant consideration for patients with HCC, who often require multiple TACE procedures for disease control and bridging to transplant [21].

The literature describing the use of Oncozene microspheres for HCC is limited, but our findings are consistent with those of previous studies. In comparison to our study, previous reports consisted of single cohort designs, and examined smaller particle sizes with higher doses of doxorubicin. Malagari et al. prospectively studied a group of 52 patients treated with 40/75/100 μm particles, depending on tumor size, loaded with either 100 or 150 mg of doxorubicin [10]. The complete and objective target lesion response rate according to the mRECIST criteria, 1 month after TACE, were 13.7% and 58.8%, respectively. The observed rate of post-embolization syndrome in this study ranged from 13 to 46%. The MIRACLE I Study examined 25 patients who were treated with 75 μm particles loaded with 150 mg of doxorubicin [9]. According to the mRECIST criteria for the best response, the reported complete response and objective response were 48% and 67%, respectively. There was a 12% rate of serious adverse events in the first 30 days, and post-embolization syndrome occurred in 50–86% of patients. Greco et al. retrospectively analyzed 48 patients who were treated with 40 μm particles loaded with 100 mg of doxorubicin [8]. The reported best complete and objective response according to the mRECIST criteria in this study were 46% and 72.6%, respectively. There were no major complications, and the incidence of post-embolization syndrome was 15%.

Limitations of this study are due to the small number of patients and retrospective design, which affects the interpretability of the survival data as stated above. There is expected risk of misclassification bias due to its retrospective nature. However, this was minimized as the data collection process was primarily performed by two investigators. Another limitation due to the retrospective nature of the study is that follow-up echocardiograms were not obtained to assess for anthracycline-related toxicity. As for the medication usage, although an attempt was made to quantify the amount of narcotics used by each patient, we cannot account for narcotics used by a patient after discharge. Many patients in this study received treatment for HCC prior to undergoing OZ-TACE or LC-TACE. These treatments could potentially affect hepatic arterial integrity and affect tumor vascularity, although this is difficult to quantify and control for. Future directions may include a prospective, comparison study to focus on a mid-term and long-term clinical, complications and safety outcomes, including post-embolization syndromes, true narcotic usage in both in-patient and out-patient settings, tumor response with and without other treatments, and potential combinational use of transarterial chemoembolization with and without systemic treatment. As clinical experience with OZ-TACE grows, outcomes among larger patient cohorts may also be assessed with more rigorous analysis, such as propensity score matching. It would also be interesting to see if clinical or radiographic outcomes varied with Oncozene microsphere size, as almost all the patients in the OZ-TACE cohort were treated with 75 μm particles.

In conclusion, the results of this study suggest that with significantly reduced doses of doxorubicin, TACE performed with Oncozene microspheres in a heterogeneous patient population is well-tolerated, safe, and produces a similar radiographic response and survival rate when compared to TACE performed with LC Bead.

## Figures and Tables

**Table 1:** Baseline clinical characteristics.

	Oncozene (n = 29)	LC (n = 29)	*p* Value
Age (y) *	67 (46–90)	65 (49–89)	0.18
Male/Female	20/9	19/10	1.00
Etiology			0.02
Hepatitis B Virus	2	3	
Hepatitis C Virus	15	19	
Nonalcoholic Steatohepatitis	10	3	
Alcohol	0	4	
Cryptogenic	2	0	
BCLC Stage			0.80
A	4	2	
B	13	15	
C	12	12	
Child–Pugh Status			0.36
A	24	20	
B	5	9	
C	0	0	
ECOG Performance Status			0.83
0	19	17	
1	9	10	
2	1	2	
Previous Treatment			
Resection	2	1	1.00
Chemotherapy	1	4	0.35
Ablation	1	6	0.10
TACE	17	12	0.29
Tumor Burden and Distribution			
Unifocal/Multifocal	9/20	9/20	1.00
Unilobar/Bilobar	18/11	15/14	0.60
Largest Lesion Diameter (cm) *	5.1 (1.4–9.5)	4.3 (1.1–13.0)	0.49
Total Sum of Enhancing Target Lesion Diameters (cm) *	4.4 (1.5–12.2)	4.1 (1.1–11.0)	0.86
Laboratory Values			
Serum aspartate aminotransferase (IU/L) *	47 (15–160)	55 (16–292)	0.39
Serum alanine aminotransferase (IU/L) *	44 (15–204)	34 (9–232)	0.15
Serum total bilirubin (mg/dL) *	0.9 (0.3–3.0)	1.1 (0.2–2.2)	0.31
Serum alkaline phosphatase (IU/L) *	118 (59–306)	117 (47–348)	0.38
Serum albumin (g/dL) *	3.8 (2.7–4.7)	3.4 (2.6–4.2)	0.008
International normalized ratio *	1.1 (1.0–1.3)	1.2 (0.9–1.6)	0.000
Serum α-fetoprotein (ng/mL) *	13.7 (2.0–12,294)	26.9 (2.1–54,450)	0.48
Chemotherapeutic Dose Delivered (mg) *	50 (17–100)	75 (25–112)	0.0005

Unless otherwise indicated, data are number of patients. BCLC = Barcelona Clinic Liver Cancer; ECOG = Eastern Cooperative Oncology Group; TACE = Transarterial Chemoembolization. * Data are medians, with ranges in parentheses.

**Table 2:** Adverse events.

	Oncozene (n = 29)	LC (n = 29)	*p* Value
Fever	5 (17)	1 (3)	0.19
Pain *	22 (76)	17 (59)	0.26
Nausea *	21 (72)	17 (59)	0.41
Postembolization Syndrome **	25 (86)	24 (83)	1.00
Daily Narcotic Requirement (mg) ‡	15 (0–131)	7.5 (0–85)	0.10
Complications			
Overall	6 (21)	3 (10)	0.47
Major	6 (21)	2 (7)	0.25
Minor	0 (0)	1 (3)	1.00

Data are number of patients, with percentage in parentheses. Unless otherwise noted, adverse events were Grade 1/2. * Three Grade 3 events in the Oncozene group, and two Grade 3 events in the LC Bead group. ** Five Grade 3 events in the Oncozene group, and two Grade 3 events in the LC Bead group. ‡ Median and range in parentheses, expressed as PO morphine equivalents [13].

**Table 3:** Change in laboratory values from the baseline.

	Oncozene (n = 29)	LC (n = 29)	*p* Value
Post-procedure Day 1			
Aspartate aminotransferase (%)	19.6 (−29.2–378.1)	14.1 (−24.1–1257.1)	0.84
Alanine aminotransferase (%)	9.1 (−54.9–373.5)	16.0 (−25.4–2022.2)	0.37
Total bilirubin (%)	0.0 (−33.3–233.3)	18.8 (−33.3–100.0)	0.05
Alkaline phosphatase (%)	−10.2 (−28.7–24.4)	−7.1 (−30.0–10.4)	0.22
Albumin (%)	−7.9 (−23.3–2.78)	−5.3 (−27.0–12.9)	0.28
Post-procedure Day 30			
Aspartate aminotransferase (%)	0.0 (−66.0–329.2)	−5.9 (−82.9–78.3)	0.22
Alanine aminotransferase (%)	−10.0 (−80.4–655.6)	−2.8 (−72.7–117.5)	0.93
Total bilirubin (%)	−16.7 (−68.4–100.0)	0.0 (−50.0–100.0)	0.42
Alkaline phosphatase (%)	15.3 (−20.0–130.2)	14.4 (−28.1–176.6)	0.60
Albumin (%)	0.0 (−26.8–37.0)	−3.5 (−21.9–25.8)	0.87
International normalized ratio (%)	0.0 (−9.1–36.4)	0.0 (−18.8–15.4)	0.009
α-fetoprotein (%)	−13.3 (−85.2–552.5)	−17.6 (−91.6–221.0)	0.86

Data are medians, with ranges in parentheses.

## Data Availability

Not acceptable.

## References

[1] Ferlay J., Soerjomataram I., Dikshit R., Eser S., Mathers C., Rebelo M., Parkin D.M., Forman D., Bray F. (2015). Cancer incidence and mortality worldwide: Sources, methods and major patterns in GLOBOCAN 2012. Int. J. Cancer.

[2] El-Serag H.B., Kanwal F. (2014). Epidemiology of hepatocellular carcinoma in the United States: Where are we? Where do we go?. Hepatology.

[3] Petruzzi P., Crocetti L., Lencioni R. (2013). Chemoembolization of Hepatocellular Carcinoma. Semin. Interv. Radiol..

[4] Lammer J., Malagari K., Vogl T., Pilleul F., Denys A., Watkinson A., Pitton M., Sergent G., Pfammatter T., On Behalf of the PRECISION V Investigators (2009). Prospective Randomized Study of Doxorubicin-Eluting-Bead Embolization in the Treatment of Hepatocellular Carcinoma: Results of the PRECISION V Study. Cardiovasc. Interv. Radiol..

[5] Gaba R.C. (2012). Chemoembolization practice patterns and technical methods among interventional radiologists: Results of an online survey. Am. J. Roentgenol..

[6] Lee K.-H., Liapi E., Vossen J.A., Buijs M., Ventura V.P., Georgiades C., Hong K., Kamel I., Torbenson M.S., Geschwind J.-F.H. (2008). Distribution of iron oxide–containing embosphere particles after transcatheter arterial embolization in an animal model of liver cancer: Evaluation with MR imaging and implication for therapy. J. Vasc. Interv. Radiol..

[7] Guiu B., Schmitt A., Reinhardt S., Fohlen A., Pohl T., Wendremaire M., Denys A., Blümmel J., Boulin M. (2015). Idarubicin-loaded ONCOZENE drug-eluting embolic agents for chemoembolization of hepatocellular carcinoma: In vitro loading and release and in vivo pharmacokinetics. J. Vasc. Interv. Radiol..

[8] Greco G., Cascella T., Facciorusso A., Nani R., Lanocita R., Morosi C., Vaiani M., Calareso G., Greco F.G., Ragnanese A. (2017). Transarterial chemoembolization using 40 µm drug eluting beads for hepatocellular carcinoma. World J. Radiol..

[9] Richter G., Radeleff B., Stroszczynski C., Pereira P., Helmberger T., Barakat M., Huppert P. (2018). Safety and feasibility of chemoembolization with doxorubicin-loaded small calibrated microspheres in patients with hepatocellular carcinoma: Results of the MIRACLE I prospective multicenter study. Cardiovasc. Interv. Radiol..

[10] Malagari K., Kiakidis T., Pomoni M., Moschouris H., Emmanouil E., Spiridopoulos T., Sotirchos V., Tandeles S., Koundouras D., Kelekis A. (2016). Pharmacokinetics, safety, and efficacy of chemoembolization with doxorubicin-loaded tightly calibrated small microspheres in patients with hepatocellular carcinoma. Cardiovasc. Interv. Radiol..

[11] Albrecht K.C., Aschenbach R., Diamantis I., Eckardt N., Teichgräber U. (2021). Response rate and safety in patients with hepatocellular carcinoma treated with transarterial chemoembolization using 40-µm doxorubicin-eluting microspheres. J. Cancer Res. Clin. Oncol..

[12] Bailey R.E., Surapaneni P.K., Core J., Vidal L.L.C., LeGout J., Ritchie C., Frey G., McKinney J.M., Sella D., Paz-Fumagalli R. (2019). Safety and efficacy of locoregional therapy for metastatic pancreatic ductal adenocarcinoma to the liver: A single-center experience. J. Gastrointest. Oncol..

[13] McAuley D. (2018). Opioid Analgesic Converter. http://www.globalrph.com/narcoticonv.htm.

[14] Brown D.B., Nikolic B., Covey A.M., Nutting C.W., Saad W.E., Salem R., Sofocleous C.T., Sze D.Y., Committee SoIRSoP (2012). Quality improvement guidelines for transhepatic arterial chemoembolization, embolization, and chemotherapeutic infusion for hepatic malignancy. J. Vasc. Interv. Radiol..

[15] U.S. Department of Health and Human Services NIoH, National Cancer Institute (2010). Common Terminology Criteria for Adverse Events (CTCAE) v4.03. https://evs.nci.nih.gov/ftp1/CTCAE/CTCAE_4.03_2010-06-14_QuickReference_5x7.pdf.

[16] Lencioni R., Llovet J. (2010). Modified RECIST (mRECIST) assessment for hepatocellular carcinoma. Semin. Liver Dis..

[17] Prajapati H.J., Spivey J.R., Hanish S.I., El-Rayes B.F., Kauh J.S., Chen Z., Kim H.S. (2013). mRECIST and EASL responses at early time point by contrast-enhanced dynamic MRI predict survival in patients with unresectable hepatocellular carcinoma (HCC) treated by doxorubicin drug-eluting beads transarterial chemoembolization (DEB TACE). Ann. Oncol..

[18] Shim J.H., Lee H.C., Kim S.-O., Shin Y.M., Kim K.M., Lim Y.-S., Suh D.J. (2012). Which response criteria best help predict survival of patients with hepatocellular carcinoma following chemoembolization? A validation study of old and new models. Radiology.

[19] Trevisani F., Magini G., Santi V., Morselli-Labate A.M., Cantarini M.C., Di Nolfo M.A., Del Poggio P., Benvegnù L., Rapaccini G., Farinati F. (2007). Impact of etiology of cirrhosis on the survival of patients diagnosed with hepatocellular carcinoma during surveillance. CME. Am. J. Gastroenterol..

[20] Padia S.A., Shivaram G., Bastawrous S., Bhargava P., Vo N.J., Vaidya S., Valji K., Harris W.P., Hippe D., Kogut M.J. (2013). Safety and efficacy of drug-eluting bead chemoembolization for hepatocellular carcinoma: Comparison of small-versus medium-size particles. J. Vasc. Interv. Radiol..

[21] Rahman A.M., Yusuf S.W., Ewer M.S. (2007). Anthracycline-induced cardiotoxicity and the cardiac-sparing effect of liposomal formu-lation. Int. J. Nanomed..

